# Hip Ankylosis after Untreated Septic Arthritis by *Escherichia coli* : A Case Report

**DOI:** 10.1055/s-0041-1736515

**Published:** 2021-11-04

**Authors:** Sabit Sllamniku, Lul Raka, Emir Q. Haxhija, Ardiana Murtezani

**Affiliations:** 1 Departamento de Ortopedia, Centro Clínico Universitário do Kosovo, Pristina, Kosovo; 2 Departamento de Microbiologia, Faculdade de Medicina, Instituto Nacional de Saúde Pública do Kosovo, Pristina, Kosovo; 3 Faculdade de Medicina, Universidade de Pristina, Pristina, Kosovo; 4 Departamento de Cirurgia Pediátrica e Adolescente, Universidade Médica Graz, Graz, Áustria; 5 Departamento de Medicina Física e Reabilitação, Centro Clínico Universitário do Kosovo, Pristina, Kosovo

**Keywords:** ankylosis, arthritis, infectious, hip joint, *Escherichia coli*

## Abstract

Septic arthritis is usually reported in elderly patients with other underlying medical conditions. Septic arthritis by *Escherichia coli* is a rare infection. We are describing the case of a 70-years old patient who presented with a suppurative fistula, limited movements of the right lower limb, and a trauma that occurred at the age of 12. Throughout this time, the fistula had been present, secreting pus. A detailed clinical investigation revealed a pyogenic infection present in the femoral epiphysis followed by an elevated sedimentation rate. After the surgical intervention, *E. coli* was isolated from the clinical samples, and the decision to place gentamicin beads within the surgical wound was taken. The patient was treated with antibiotics. Four months after the intervention, the suppurative fistula was completely healed.

Later on, the patient was no longer interested anymore in continuing with the treatment plan. As he refused to remove the gentamicin chain beads and the hip endoprosthesis, he was subsequently referred to the primary care clinic for conservative management and follow-up. He walked with a limp wearing orthopedic shoes and not using crutches or any other type of walking-aid. Four years after the surgical intervention, the gentamicin chain beads are still within the bone. Septic arthritis caused by *E. coli* can remain active for decades, secreting pus and self-isolating. Prompt diagnosis, adequate surgical intervention, and antimicrobial therapy are essential for the treatment.

## Introduction

In adults presenting with acute monoarticular arthritis, septic arthritis. which is usually located in the knee and hip joints, is a key consideration. 1 2 Delays in the prescription of the appropriate antibiotic therapy within the first 48 hours of the onset of symptoms can result in subchondral bone loss and permanent joint dysfunction. 1

Many bacterial isolates have been reported in the etiology of septic arthritis. The most common etiology is *Staphylococcus aureus* , which is responsible for 37% to 65% of the cases, depending on the geographic distribution, the incidence of comorbid rheumatic disease, and the proportion of infections involving the joints. There has been an increase in joint conditions caused by methicillin-resistant *S. aureus* (MRSA), particularly in the elderly and on patients recently submitted to orthopedic surgery. Gram-negative bacilli account for ∼ 5% to 20% of the cases. The most common Gram-negative organisms are *Pseudomonas aeruginosa* and *Escherichia coli* , usually in patients with a history of use of intravenous drugs, neonates, the elderly, and immunocompromised patients. 1 2 3 4

The independent risk factors in cases of infectious arthritis include bacteriuria, hip joint involvement, and use of steroids. Advanced age and frailty, compromised immunity, skin infections, recurrent urinary tract infection, and recent abdominal surgery are previously known risk factors for infectious arthritis. However, the evidence is still unclear. 4 5

The present paper aims to describe a case of a long-lasting fistulous suppurative hip arthritis without previous infections or disease caused by *E. coli* .

## Case Report

**Fig. 1:**
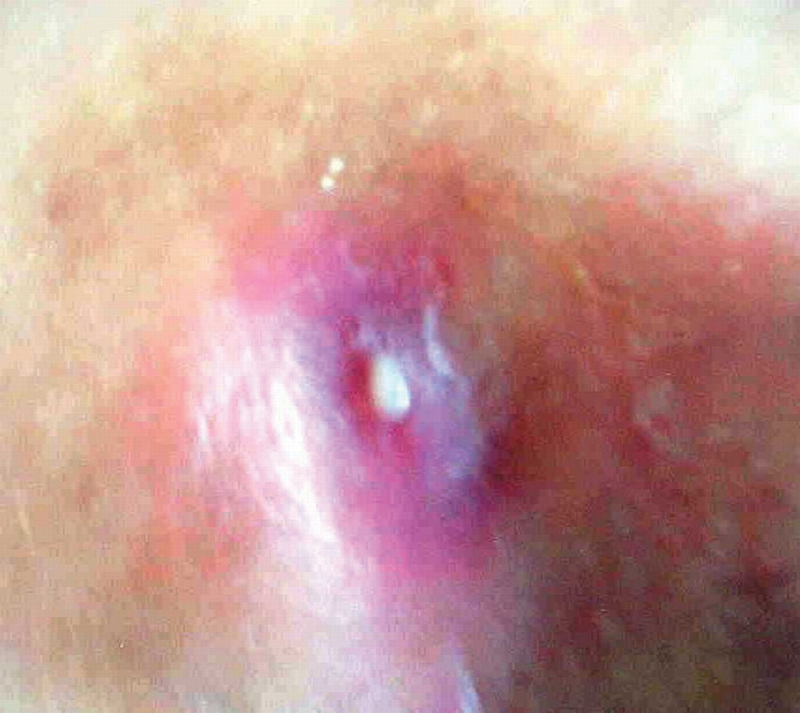
Suppurative fistula from hip arthritis.

A 70-year-old retired man, weighting 80 kg, non-smoker, non-alcoholic, without any concomitant diseases and previous surgeries. He worked as a security guard for an elementary school, and was admitted to the orthopedic and traumatology clinic due to a suppurative fistula and redness around the region of the greater trochanter of the right femur ( Fig. 1 ).

**Fig. 2:**
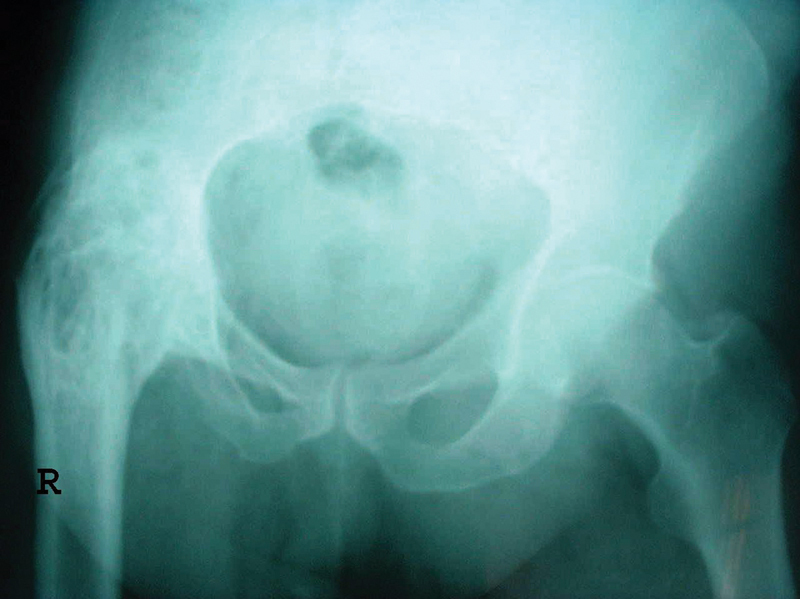
Simple radiography of the pelvis, showing ankylosis of the right hip.

He reported having no history of pain and fever. The movements of the right hip joint were limited to 30° of flexion, 10° of abduction, and no rotation. The patient limped while walking due to a shortening of the leg of ∼ 5 cm. According to his life history, at the age of 12, he suffered blunt trauma from a crash. For 58 consecutive years, the patient reports that the fistula has been present on his limb, secreting pus. Upon admission to the hospital, plain radiography and X-ray fistulography revealed a focus of infection focus in the femoral epiphysis ( Fig. 2 ).

**Fig. 3:**
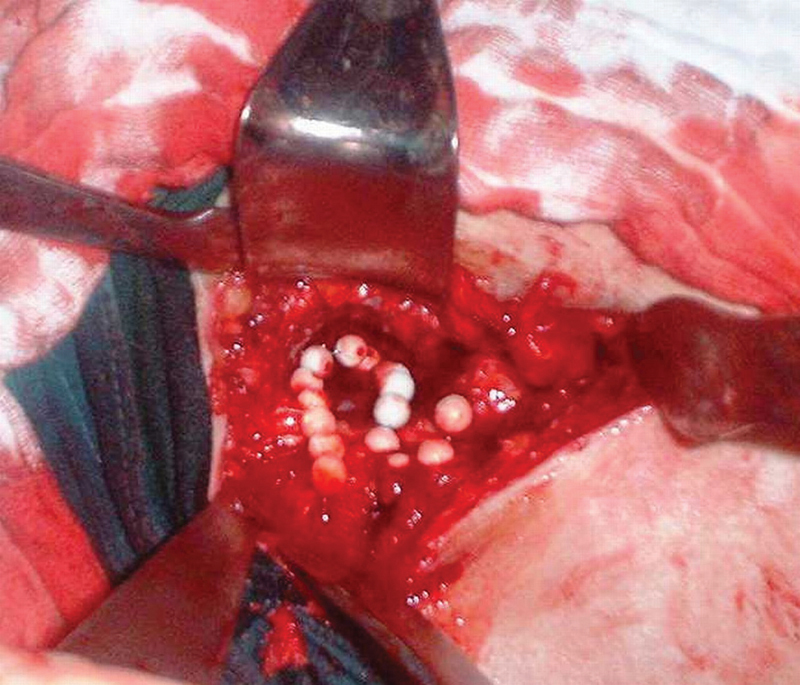
Enlarged bone fistula filled with 15 gentamicin chain beads.

Laboratory investigations showed a slightly elevated sedimentation rate (22/45), and the result of C-reactive protein (CRP) test was 12 mg/l. The results of further respiratory, digestive, and urinary tract investigations were completely normal, and did not point to any concomitant disease or illness. The urinalysis also presented average values. After a clinical examination and laboratory evaluation, in February 2017, surgical intervention was performed under spinal anesthesia. From the sample taken within the bone cavity by curettage, *E. coli* were isolated from the purulent foci as the causative bacteria. This *E. coli* isolate was sensitive to third-generation cephalosporins, gentamicin, and fluoroquinolones, but resistant to amoxicillin and tetracycline. After removal of the skin and bone fistula and irrigation, a total of 15 gentamicin chain beads were introduced ( Fig. 3 ).

**Fig. 4:**
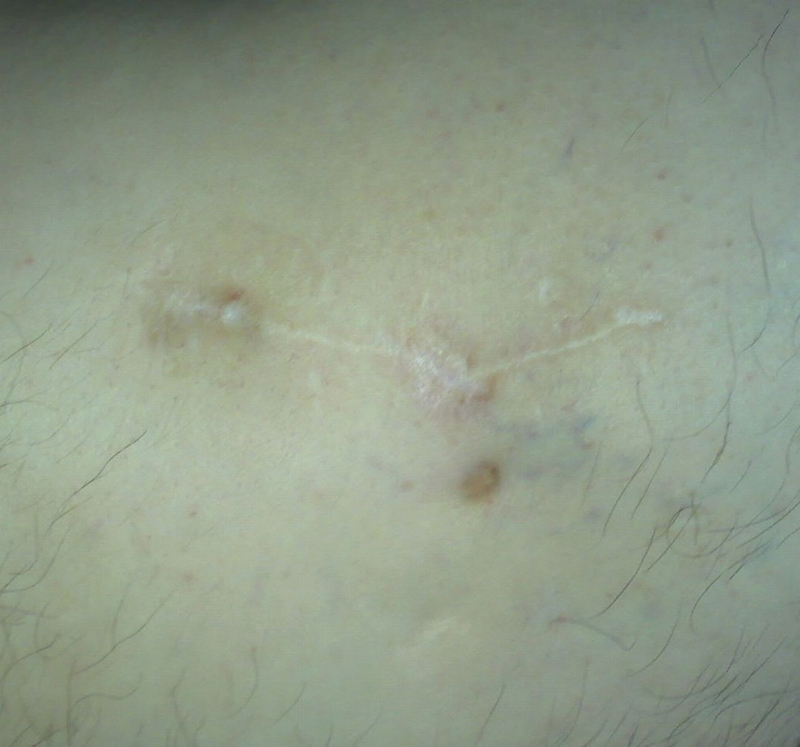
Postoperative scar in the region of the great trochanter without fistula and redness.

**Fig. 5:**
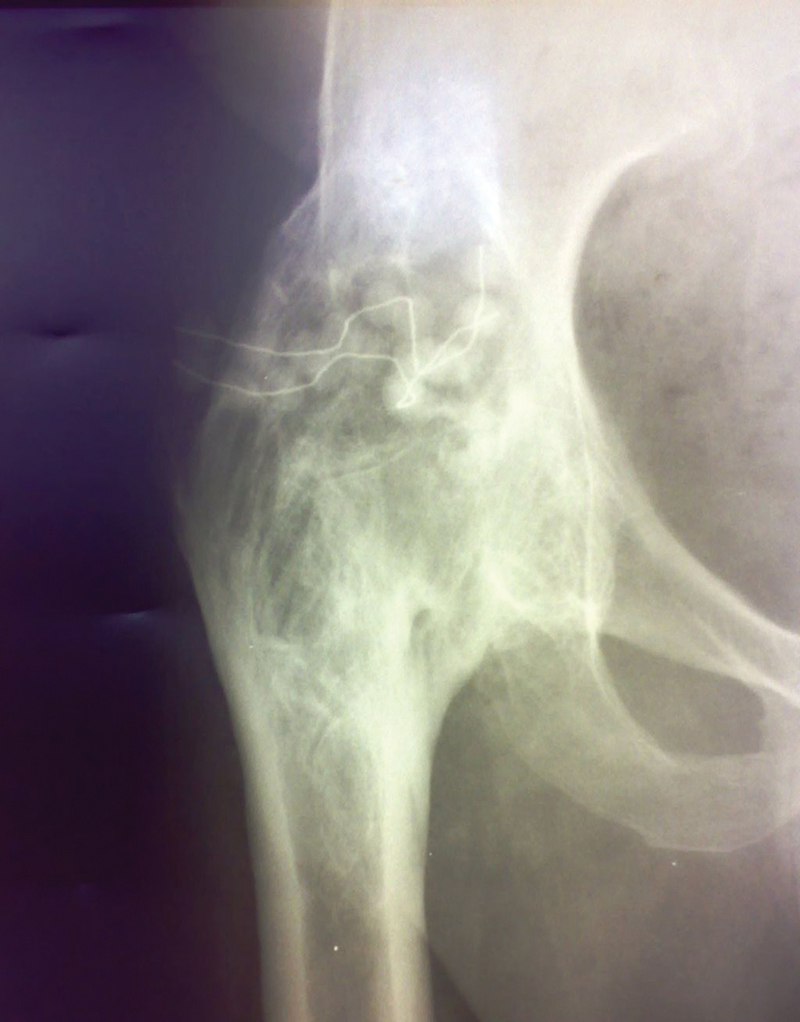
A plain radiograph of the right hip showing the containment of the gentamicin beads.

The drain was removed 48 hours after the intervention. The patient was treated with ceftriaxone 2 g during the intervention, ceftriaxone 2 g b.i.d. for the first 3 weeks after the intervention, and ciprofloxacin 750 mg b.i.d for another 3 weeks. Four months after the intervention, the suppurative fistula was completely healed. Blood sedimentation rates decreased to 18/30, and the CRP test was negative. Nine months after the intervention, sedimentation rates fell to 6/12, the and CRP test was negative. One year after the surgical management, considering the normalization of the laboratory results, plain radiography, clinical condition, and our patient's best interest, we suggested continuing with the treatment plan and removing the gentamicin chain beads and the hip endoprosthesis. The patient refused because of his satisfaction with the results of the intervention. He walked with a limp wearing orthopedic shoes, without using crutches or any other type of walking aid. As we care greatly for our patients, we honored our patient's autonomy to decide, and subsequently referred him for conservative management and follow-up. Four years after the surgical intervention, the gentamicin chain beads are still within the bone ( Figs. 4 and 5 ). No side effects such as redness, swelling, heat, ototoxicity, or nephrotoxicity have been reported.

## Discussion

Septic arthritis is an acute infection of the joint that occurs most commonly in young children; it is mainly monoarticular, and is frequently located in the knee and hip joints. 2 The incidence of septic arthritis ranges widely, between 4 and 29 cases per 100 thousand inhabitants/year. The most common route of entry into the joint is hematogenous spread during bacteremia. Pathogens may also enter through direct inoculation (for example: arthrocentesis, arthroscopy, trauma) or spread continuously due to local infections (such as osteomyelitis, septic bursitis, abscess). 1

Despite numerous independent risk factors, 4 5 hip infection by *E. coli* can occur without any previous history of disease.

The outcome in patients with septic arthritis due to some of the more virulent organisms, such as superantigen-producing *S. aureus* and certain Gram-negative bacilli, is poor, despite an optimal therapy. Evacuation of purulent material with surgical methods is necessary, and then antibiotics are adjusted based on the results of culture and sensitivity. Adequate drainage of the joint is the preferred method of intervention. 1 2 3

In addition to surgery, and the intravenous and oral administration of antibiotics, our treatment method is also performed with the local application of antibiotics in the form of gentamicin beads. 6

We did not consider the option of a Girdlestone-type resection during the first procedure because the patient reported no pain. This procedure would have shortened more the length of the leg and increased the asymmetric abnormality of the gait, making walking more difficult and the tendency to limp, more obvious and emphasized. 7

*E. coli* is rarely the causative organism of acute suppurative arthritis. Many reviews of the literature on acute suppurative arthritis do not mention the role of *E. coli* . Furthermore, even in the nonsuppurative form, arthritis is an uncommon manifestation of *E. coli* septicemia. 8

Septic arthritis caused by the bacterium *E. coli* can remain active for decades by secreting and self-isolating. Prompt diagnosis, adequate surgical intervention, and antimicrobial therapy are essential in the treatment, even in long-lasting infections. Complications regarding the joint, such as ankylosis, may occur.
