# Supplementary material for: Hip Ankylosis after Untreated Septic Arthritis by Escherichia coli : A Case Report
Source: Rev Bras Ortop (Sao Paulo). 2021 Nov 4;59(Suppl 1):e22–5. doi: 10.1055/s-0041-1736515 (PMC11254424; doi:10.1055/s-0041-1736515)
Supplement: Related texts

# Research article: Anquilose do quadril após artrite séptica por Escherichia coli não tratada: Relato de caso

**Keywords:** anquilose, articulação do quadril, artrite infecciosa, *Escherichia coli*

## Abstract

A artrite séptica é geralmente relatada em pacientes idosos com outras condições médicas subjacentes. Artrite séptica por *Escherichia coli* é uma infecção rara. Descrevemos o caso de um paciente de 70 anos que apresentou uma fístula supurativa, movimentos limitados do membro inferior direito, e um trauma ocorrido aos 12 anos de idade. Durante todo esse tempo, a fístula esteve presente, secretando pus. Uma investigação clínica detalhada revelou uma infecção piogênica presente na epífise femoral seguida de uma taxa elevada de sedimentação. Após a intervenção cirúrgica, *E. coli* foi isolada das amostras clínicas, e tomou-se a decisão de colocar pérolas de gentamicina dentro da ferida cirúrgica. O paciente foi tratado com antibióticos. Quatro meses após a intervenção, a fístula supurativa foi completamente curada.

Mais tarde, o paciente não estava mais interessado em continuar com o plano de tratamento. Como ele se recusou a remover as pérolas da cadeia de gentamicina e a endoprótese de quadrile, ele foi posteriormente encaminhado para a clínica de atenção primária para manejo conservador e acompanhamento. Ele andava mancando usando sapatos ortopédicos, mas sem muletas ou qualquer outro tipo de dipositivo de auxílio para caminhar. Quatro anos após a intervenção cirúrgica, as pérolas da cadeia de gentamicina ainda estão inseridas no osso. A artrite séptica causada por *E. coli* pode ficar ativa por décadas, secretando pus e se isolando. Diagnóstico rápido, intervenção cirúrgica adequada, e terapia antimicrobiana são essenciais para o tratamento.

## Introdução

Em adultos que apresentam artrite monoarticular aguda, é fundamental considerar a artrite séptica, que geralmente se localiza nas articulações do joelho e do quadril. 1 2 Atrasos na prescrição de antibióticos adequados nas primeiras 48 horas de início dos sintomas podem resultar em perda óssea subcondral e disfunção articular permanente. 1

Muitos isolados bacterianos foram relatados na etiologia da artrite séptica. A etiologia mais comum é o *Staphylococcus aureus* , responsável por 37% a 65% dos casos, dependendo da distribuição geográfica, da incidência da doença reumática comórbida, e da proporção de infecções envolvendo articulações. Há um aumento nas afecções articulares causadas por *S. aureus* resistente à meticilina ( *methicillin-resistant* S. aureus, MRSA, em inglês), particularmente em idosos e pacientes recentemente submetidos a cirurgia ortopédica. Os bacilos Gram-negativos representam de 5% a 20% dos casos. Os organismos Gram-negativos mais comuns são *Pseudomonas aeruginosa* e *Escherichia coli* , geralmente em pacientes com histórico de uso de drogas intravenosas, recém-nascidos, idosos, e pacientes imunodeficientes. 1 2 3 4

Os fatores de risco independentes em casos de artrite infecciosa incluem bacteriúria, envolvimento articular do quadril, e uso de esteroides. Idade avançada e fragilidade, imunidade comprometida, infecções de pele, infecção do trato urinário recorrente, e cirurgia abdominal recente são fatores de risco previamente conhecidos para artrite infecciosa. No entanto, as evidências ainda não estão claras. 4 5

Este artigo tem como objetivo apresentar um caso de artrite de quadril supurativa de longa duração sem infecções anteriores ou doenças causadas por *E. coli* .

## Relato de caso

Um aposentado de 70 anos, que pesava 80 kg, não fumante, não alcoólatra, sem qualquer doença concomitante e cirurgias anteriores. Ele trabalhava como segurança de uma escola primária, e foi internado na clínica de ortopedia e traumatologia devido a uma fístula com secreção e vermelhidão em volta da região do trocânter maior do fêmur direito ( Fig. 1 ).

**Fig. 1:** Fístula supurativa por artrite do quadril.

Ele relatou não ter histórico de dor e febre. Os movimentos da articulação do quadril direito estavam limitados a flexão de 30°, abdução de 10°, e sem rotação. O paciente mancava enquanto caminhava devido ao encurtamento da perna de ∼ 5 cm. De acordo com seu histórico de vida, aos 12 anos, ele sofreu um trauma contundente em um acidente. Por 58 anos consecutivos, o paciente relata que a fístula esteve presente em seu membro, secretando pus. Ao dar entrada no hospital, a radiografia simples e a fistulografia por raios-X revelaram foco de infecção localizado na epífise femoral ( Fig. 2 ).

**Fig. 2:** Radiografia simples de bacia, evidenciando anquilose do quadril direito.

As investigações laboratoriais mostraram uma taxa de sedimentação ligeiramente elevada (22/45), e o resultado do exame dos níveis de proteína C-reativa (PCR) foi de 12 mg/l. Outras investigações respiratórias, digestivas, e do trato urinário eram resultados completamente normais, e não apontaram nenhuma doença concomitante. A urinálise também apresentou valores médios. Após exame clínico e avaliação laboratorial, em fevereiro de 2017, foi realizada intervenção cirúrgica sob anestesia espinhal. Da amostra colhida dentro da cavidade óssea pela curetagem, *E. coli* foram isolados dos focos purulentos como bactérias causais, e esse isolado foi sensível a cefalosporinas de terceira geração, gentamicina e fluoroquinolones, mas resistente à amoxicilina e tetraciclina. Após a remoção da fístula da pele e do osso, e de irrigação, foram introduzidas 15 pérolas de cadeia de gentamicina ( Fig. 3 ).

**Fig. 3:** Fístula óssea ampliada preenchida com 15 pérolas de cadeia de gentamicina.

O dreno foi removido 48 horas após a intervenção. O paciente foi tratado com ceftriaxona 2 g durante a intervenção, ceftriaxona 2 g 2 vezes ao dia nas primeiras 3 semanas após a intervenção, e ciprofloxacino 750 mg 2x vezes ao dia por mais 3 semanas. Quatro meses após a intervenção, a fístula serosa foi completamente curada. As taxas de sedimentação sanguínea diminuíram para 18/30, e o teste de PCR foi negativo. Nove meses após a intervenção, as taxas de sedimentação caíram para 6/12, e o teste de PCR continuou negativo. Um ano após o manejo cirúrgico, considerando a normalização dos resultados laboratoriais, a radiografia simples, a condição clínica, e o melhor interesse do paciente, sugerimos continuar com o plano de tratamento e remover as pérolas da cadeia de gentamicina e a endoprótese do quadril. O paciente recusou, por sua satisfação com os resultados da intervenção. Ele andava mancando usando sapatos ortopédicos, sem usar muletas ou qualquer outro tipo de auxílio. Como cuidamos muito bem de nossos pacientes, honramos a autonomia do nosso paciente para decidir e, posteriormente o encaminhamos para gestão conservadora e acompanhamento. Quatro anos após a intervenção cirúrgica ser realizada, as pérolas da cadeia de gentamicina ainda estão dentro do osso ( Figs. 4 e 5 ). Nenhum efeito colateral, como vermelhidão, inchaço, calor, otoxicidade, ou nefrotoxicidade foi relatado até o momento.

**Fig. 4:** Cicatriz pós-operatória na região do trocânter maior sem fístula e vermelhidão.

**Fig. 5:** Radiografia simples do quadril direito mostrando a contenção das pérolas de gentamicina.

## Discussão

A artrite séptica é uma infecção aguda da articulação que ocorre mais comumente em crianças pequenas; é principalmente monoarticular, e está frequentemente localizada nas articulações do joelho e do quadril. 2 A incidência de artrite séptica tem uma ampla gama, entre 4 e 29 casos por 100 mil habitantes/ano. A rota mais comum de entrada na articulação é a propagação hematógena durante a bacteremia. Os patógenos também podem entrar por inoculação direta (por exemplo: por artrose, artroscopia, trauma) ou se propagar continuamente por infecções locais (por exemplo: osteomielite, bursite séptica, abscesso). 1

Apesar de inúmeros fatores de risco independentes, 4 5 infecções de quadril por *E. coli* podem ocorrer sem qualquer histórico anterior da doença.

Devido a alguns organismos mais virulentos, como *S. Aureus* produtor de superantígeno e certos bacilos Gram-negativos, o desfecho em pacientes com artrite séptica é ruim, apesar da ótima terapia. A limpeza de material purulento por métodos cirúrgicos é necessária, e então os antibióticos são ajustados com base nos resultados de cultura e sensibilidade. A drenagem adequada da articulação é um método preferencial de intervenção. 1 2 3

Além da cirurgia, da administração intravenosa e oral dos antibióticos, em nosso tratamento também realizamos a aplicação local de antibióticos na forma de pérolas de gentamicina. 6

Não consideramos a opção de uma ressecção do tipo Girdlestone durante o primeiro procedimento porque o paciente não relatou dor. Este procedimento teria encurtado mais o comprimento da perna e aumentado a anormalidade assimétrica da marcha, o que tornaria a caminhada mais difícil, e a tendência de mancar, mais óbvia e enfatizada. 7

*E. coli* raramente é o organismo causador da artrite supurativa aguda, e muitas revisões da literatura sobre artrite supurativa aguda não mencionam o seu papel. Além disso, mesmo na forma não supurativa, a artrite é uma manifestação incomum da septicemia por *E. coli.* 8

A artrite séptica causada pela bactéria *E. coli* pode ficar ativa por décadas, secretando e se isolando. Diagnóstico imediato, intervenção cirúrgica adequada e terapia antimicrobiana são essenciais no tratamento, até mesmo em casos de infecção de longa duração. Complicações da articulação, como a anquilose, podem ocorrer.
